# Integration of Brain Proteomes and Genome-Wide Association Data Identifies GLO1 as a Candidate Causal Gene and Therapeutic Target for Restless Legs Syndrome

**DOI:** 10.3390/ijms27104446

**Published:** 2026-05-15

**Authors:** Lingyu Zhang, Qianqian Jin, Ruochen Du, Yuxiang Liang

**Affiliations:** 1 Shanxi Key Laboratory of Human Disease and Animal Models, Experimental Animal Center of Shanxi Medical University, Taiyuan 030001, China; zhanglingyu@sxmu.edu.cn; 2 Department of Forensic Pathology, Shanxi Medical University, Taiyuan 030001, China; qianqian.jin@sxmu.edu.cn

**Keywords:** restless legs syndrome, *GLO1*, proteome-wide association study, Mendelian randomization, drug target

## Abstract

Restless legs syndrome (RLS) is a common sensorimotor disorder with limited treatment options and incompletely understood pathophysiology. Genome-wide association studies have identified numerous risk loci, but translating these findings into causal genes and therapeutic targets remains challenging. We performed a proteome-wide association study (PWAS) integrating RLS genome-wide association study (GWAS) data from FinnGen with two brain pQTL datasets (ROSMAP and Banner). We validated the identified proteins using TWAS, SMR, and colocalization analyses using brain pQTL and eQTL datasets. To further investigate peripheral protein associations, we performed SMR using plasma pQTL data from the UK Biobank Pharma Proteomics Project (UKB-PPP). We also conducted a phenome-wide association study (PheWAS) to screen for potential off-target effects of the prioritized genes, followed by drug prediction using DSigDB and molecular docking. PWAS identified *GLO1*, along with *GRWD1* and *MAP2K5*, as significantly associated with RLS. *GLO1* was identified by brain-based SMR (*p* = 0.0001), colocalization (PP.H4 = 0.96), TWAS (*p* = 0.048), and was confirmed by plasma-based SMR (*p* = 3.16 × 10^−9^) as the only protein associated with RLS. PheWAS analysis, without associations for 783 non-RLS phenotypes, confirmed the specificity of *GLO1*. Among 27 predicted *GLO1*-targeting compounds, Gambierol had the strongest binding affinity (−8.3 kcal/mol). This proteogenomic study identifies *GLO1* as a prioritized causal gene and promising drug target for RLS, combining brain and plasma data to provide new insights into pathogenesis and candidate drug development.

## 1. Introduction

Restless legs syndrome (RLS) is a common sensorimotor disorder marked by an irresistible urge to move the legs, often accompanied by uncomfortable sensations that worsen at rest and at night and ease with movement [1,2]. Affecting 5–10% of European populations, RLS disrupts sleep, mood, and daily functioning, and severe cases are linked to depression, cardiovascular disease, and suicidal ideation [3,4,5]. Current treatments are mainly dopaminergic, α2-δ ligands, and opioids that offer only temporary relief, as well as side effects such as increased dependence and tolerance [6,7]. Despite its clinical significance, the pathophysiology of RLS remains poorly understood, although evidence suggests brain iron deficiency, dopaminergic dysfunction, abnormalities in other neurotransmitters, and a genetic predisposition [8,9,10]. The substantial genetic contribution to RLS is well established. Family studies have demonstrated substantial heritability, with up to 60% of idiopathic cases reporting a positive family history [11]. Recently, genome-wide association studies have identified more than 160 risk loci involved in neurodevelopment, synaptic plasticity, and iron homeostasis [12,13]. Despite this progress, most risk variants reside in non-coding regions, leaving causal genes and pathways to be elucidated.

Translating these genetic associations into functional understanding requires integrative approaches that bridge GWAS with molecular quantitative trait loci. Proteome-wide association study (PWAS) integrates GWAS summary statistics with protein quantitative trait locus (pQTL) data to identify proteins whose genetically predicted abundances are associated with disease risk [14]. Unlike transcriptome-wide association studies (TWAS), PWAS directly assesses the effector molecules—proteins—that are more proximal to disease mechanisms and represent direct drug targets [15]. This approach is particularly well-suited for neurological disorders like RLS, where brain-specific protein regulation may hold the key to disease mechanisms [16,17]. Brain pQTL data derived from dorsolateral prefrontal cortex tissues from the ROSMAP and Banner cohorts provide an opportunity to examine protein-level associations with neurological traits systematically [14,18]. Given the central role of the brain in RLS pathogenesis, integrating brain pQTL data with RLS GWAS offers a powerful strategy to pinpoint causal proteins and prioritize therapeutic targets. Previous Mendelian randomization studies have identified plasma proteins such as *MAN1A2* as potential RLS drug targets [19]. However, plasma proteomic profiles may not fully reflect brain-specific processes relevant to RLS.

This study represents a systematic proteome-wide investigation of RLS, integrating brain pQTL data with RLS GWAS to uncover causal genes and evaluate their druggability through multi-omics validation and functional analyses. We further validated our findings using TWAS (CMC brain eQTL), summary-based Mendelian randomization (SMR), and Bayesian colocalization analyses. To validate and assess the generalizability of our brain-derived findings, we performed SMR analysis using plasma pQTL data from the UK Biobank (UKB-PPP). Finally, we predicted candidate drugs targeting the prioritized genes and evaluated their binding affinities using molecular docking.

## 2. Results

### 2.1. PWAS Identified Three Candidate Genes Associated with RLS Using Brain pQTLs

**Figure 1:**
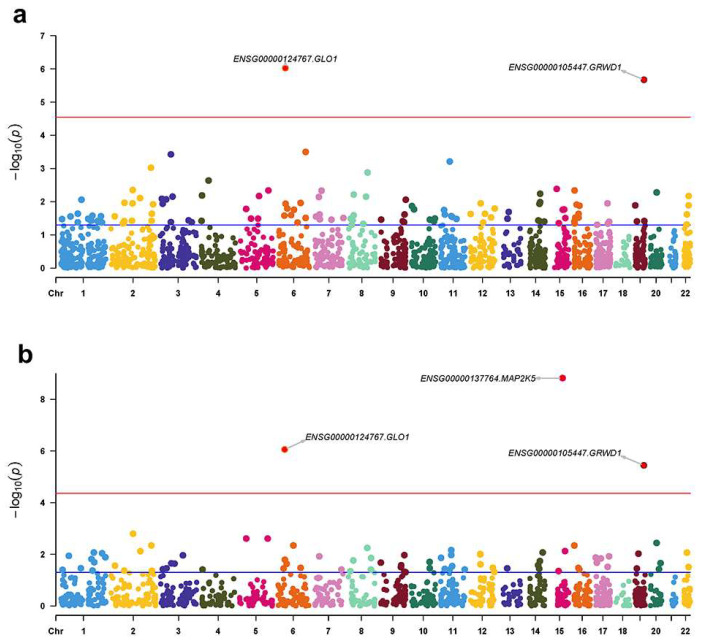
Manhattan plots of significant human brain proteins identified in the proteome-wide association study (PWAS). (**a**) Discovery PWAS using ROSMAP brain pQTL (*n =* 376) and FinnGen RLS GWAS (*n =* 500,348). (**b**) Confirmation PWAS using Banner brain pQTL (*n =* 152) and FinnGen RLS GWAS (*n =* 500,348). Each point represents a gene, with its genomic position along the *x*-axis and the strength of its genetically predicted protein-RLS association on the *y*-axis (−log_10_ *p*-value). The red dashed line indicates the Bonferroni-corrected significance threshold. Genes surpassing this threshold are highlighted.

To identify brain proteins associated with RLS, we performed PWAS integrating RLS GWAS (FinnGen) with two independent brain pQTL datasets (discovery: ROSMAP, *n =* 376; validation: Banner, *n =* 152). In the discovery PWAS, two genes surpassed FDR significance: *GLO1* (Z = −4.90, P = 9.45 × 10^−7^, *p* adjust = 0.0017) and *GRWD1* (Z = −4.74, P = 2.13 × 10^−6^, *p* adjust = 0.0037), both with negative z scores indicating decreased RLS risk (Figure 1a, Table 1 and Table S1). Both replicated in the validation PWAS (*GLO1*: Z = −4.92, P = 8.68 × 10^−7^, *p* adjust = 0.0010; *GRWD1*: Z = −4.63, P = 3.59 × 10^−6^, *p* adjust = 0.0041), and *MAP2K5* additionally emerged as a significant risk-increasing protein (Z = 6.04, P = 1.50 × 10^−9^, *p* adjust = 1.72 × 10^−6^) (Figure 1b, Table 1 and Table S2). Thus, three candidate genes (*GLO1*, *GRWD1*, and *MAP2K5*) were prioritized for further analyses.

### 2.2. SMR, Colocalization and TWAS Analyses Confirmed One Brain-Expressed Genes Associated with RLS

To validate the PWAS findings, we performed SMR using brain eQTL data (CMC). *GLO1* showed a significant SMR association (OR = 0.060, 95%CI: 0.014–0.248, *p* = 0.0001, *p* HEIDI = 0.983), with colocalization supporting a shared causal variant (PP.H4 = 0.96) and TWAS confirming the association (Z = −1.977, *p* = 0.048). *GRWD1* was also significant in SMR (OR = 0.373, 95%CI: 0.184–0.755, *p* = 0.0062, *p* HEIDI = 0.343) and TWAS (Z = −3.464, *p* = 5.32 × 10^−4^), though colocalization evidence was weaker (PP.H4 = 0.52). *MAP2K5* was significant only in TWAS (Z = 4.388, *p* = 1.15 × 10^−5^), with no available SMR or colocalization data. Thus, among the three prioritized genes, only *GLO1* was consistently supported as a protective factor across all brain-based analytical frameworks, while *GRWD1* and *MAP2K5* showed only partial or single-method evidence (Table 2).

### 2.3. SMR Analysis Revealed 1 Genes Associated with RLS Pathogenesis in Plasma

To test whether peripheral proteins contribute to RLS, we performed SMR using plasma pQTL data from UKB-PPP. Only *GLO1* survived Bonferroni correction (OR = 0.677, 95%CI: 0.596–0.771, *p* SMR = 3.16 × 10^−9^, *p* adjust = 5.27 × 10^−6^, *p* HEIDI = 0.999) (Table 3 and  Table S3). The association was supported by an independent RLS GWAS (OR = 0.808, 95%CI: 0.753–0.868, *p* SMR = 3.16 × 10^−6^) and by blood eQTL data (eQTLgen: OR = 0.726, 95%CI: 0.639–0.826, *p* SMR = 1.12 × 10^−6^) (Table 3). These results identify *GLO1* as the only plasma protein consistently associated with RLS, with higher predicted abundance conferring lower risk.

### 2.4. Phenome-Wide Association Analysis

To evaluate the specificity of *GLO1* as a therapeutic target, we performed a PheWAS using its two independent cis-pQTLs: rs1781735 (derived from a brain pQTL) and rs4746 (derived from a plasma pQTL). After Bonferroni correction, neither SNP showed a significant association with any of the 783 non-RLS phenotypes in the UK Biobank (all *p* adjusted > 0.05) (Supplementary Tables S4 and S5). The absence of pleiotropic associations supports genetic specificity of *GLO1* as a potential drug target for RLS.

### 2.5. Candidate Drug Prediction

Using the DSigDB database, 27 chemical compounds were predicted to be potentially effective intervention drugs (Table 4 and Table S6). 2-mercaptoethanol (TTD 00000601) had the lowest *p*-value.

### 2.6. Molecular Docking

**Figure 2:**
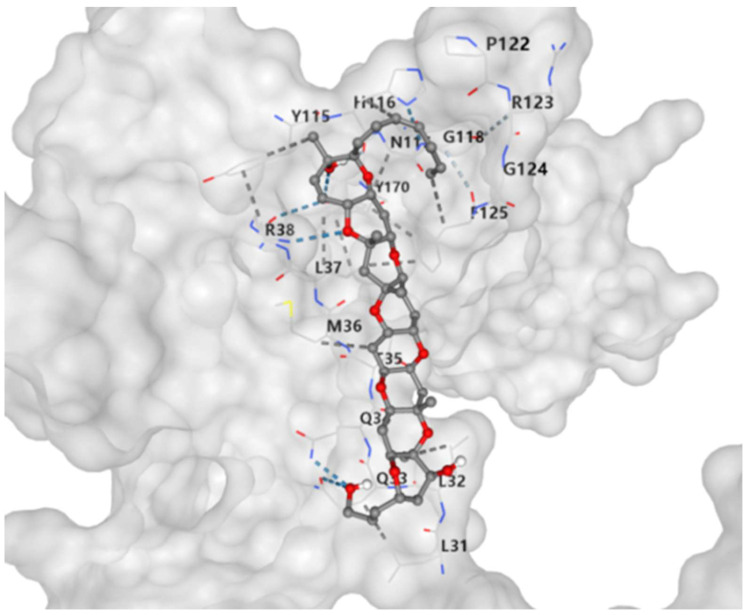
Molecular docking results of *GLO1* and the compound Gambierol (CTD 00003965).

Molecular docking was used to evaluate the binding affinity between drug candidates and their targets and to identify druggable targets. AutoDock Vina was used to identify binding sites and interactions for all 27 drug candidates with *GLO1*, and to calculate the binding energy for each interaction (Table 4 and Table S6). Except for antimony (an atom), all drug candidates formed hydrogen bonds and electrostatic interactions with their targets. Gambierol showed the strongest binding affinity (−8.3 kcal/mol) among the 27 docked compounds, followed by kaempferol (−7.5 kcal/mol) (Figure 2).

The binding pocket corresponds to CurPocket C1, the largest cavity predicted by CB-Dock2 (grid box size: 14 × 14 × 14 Å; center coordinates: 0, 5, 0). The three-dimensional structure of human *GLO1* was obtained from the AlphaFold Protein Structure Database (AF-Q04760-F1). Docking was performed using AutoDock Vina (version 1.2.0) as implemented in CB-Dock2. The docking pose and interacting residues (e.g., P122, R123, Y115, N11, G118, G124, F125, R38, L37, M36, Q3, Q32, L31, L32, L35) were visualized directly within the CB-Dock2 server using NGL Viewer. Hydrogen bonds are indicated by dashed lines, and hydrophobic interacting residues are shown as surface patches.

## 3. Discussion

In this study, we integrated brain proteome and transcriptome data with RLS GWAS data to identify drug targets implicated in RLS. Using multi-stage PWAS, SMR, colocalization, and TWAS analyses, we found *GLO1*, *GRWD1*, and *MAP2K5* associated with RLS. Plasma pQTL-based SMR further supported *GLO1* as the only protein consistently across peripheral datasets. *GLO1* demonstrated target specificity in PheWAS and druggability potential, with Gambierol identified as a high-affinity ligand by molecular docking. However, Gambierol is a known marine neurotoxin produced by *Gambierdiscus toxicus* and associated with ciguatera fish poisoning [21], exerting potent neurotoxicity by blocking voltage-gated potassium channels (Kv) [22] and allosterically modulating TRPV1 channels [23]. Despite its toxicity, Gambierol offers a structural scaffold for future optimization, whereas kaempferol, a naturally occurring flavonoid with a well-documented safety profile, also binds *GLO1* favorably (−7.5 kcal/mol) and may represent a more translatable lead compound. In addition to *GLO1*, we identified *GRWD1* and *MAP2K5* as brain-expressed genes associated with RLS. *GRWD1*, which encodes a protein involved in chromatin regulation and cell cycle progression [24], showed consistent associations across PWAS, SMR, and TWAS. However, the weaker colocalization evidence (PP.H4 = 0.52) suggests that it may be influenced by linkage disequilibrium. *MAP2K5*, which encodes a key mediator of *BDNF/TrkB* signaling [25], showed significance only in TWAS, suggesting possible post-transcriptional regulation or insufficient power in current brain pQTL data. Consequently, *GLO1* remains the only gene supported by convergent evidence across all analytical layers, whereas *GRWD1* and *MAP2K5* should be regarded as suggestive candidates requiring further validation. While this warrants further study, *GLO1* emerges as the strongest genetically supported therapeutic candidate based on convergent multi-tissue evidence. However, functional validation studies are needed to confirm its causal role in RLS pathogenesis.

A recent study by Qian et al. used MR to identify *MAN1A2* as a protective plasma protein for RLS [19]. Our study complements these findings, but with a distinct focus: while Qian et al. examined plasma pQTL data exclusively, we prioritized brain-derived pQTL and eQTL datasets. Given that brain-based evidence offers more direct insight into the pathophysiology of RLS, the convergence of brain and plasma findings suggests that *GLO1* may exert systemic effects relevant to RLS, whereas *MAN1A2* may be more tissue-specific.

*GLO1* (glyoxalase I) encodes a key enzyme in the detoxification of methylglyoxal (MG), a highly reactive dicarbonyl byproduct of glycolysis [26]. MG accumulation promotes oxidative stress, advanced glycation end-product (AGE) formation [27], and inflammation [28], and this mechanism may be particularly relevant given that iron deficiency, a well-established risk factor for the disorder, not only increases oxidative stress but also directly impairs glyoxalase activity [29]. Iron deficiency increased the oxidative burden and reduced the capacity to detoxify MG, leading to disruption of dopaminergic signaling and, consequently, contributing to RLS susceptibility [30]. Our findings support the hypothesis that *GLO1*-mediated MG detoxification protects against RLS by preserving dopaminergic function and reducing oxidative stress. The identification of *GLO1* as a protective factor addresses a critical gap in RLS therapeutics, and these exploratory computational predictions further suggest that *GLO1* is a tractable target for small-molecule modulation.

Current treatments, such as dopaminergic agents, α2-δ ligands, and opioids, only provide symptomatic relief and do not modify disease progression and carry risks of augmentation, tolerance, and dependence [31,32]. By targeting oxidative stress and metabolic dysfunction, *GLO1*-based therapy could complement existing treatments or provide an alternative for patients who develop complications from long-term dopaminergic therapy [12,33]. Furthermore, given the role of oxidative stress in RLS augmentation [34], *GLO1* activators might be particularly beneficial in preventing or managing this challenging clinical phenomenon.

Our study has several strengths, including multi-layered validation (PWAS, SMR, colocalization, and TWAS) to ensure validation, and integration of brain and plasma pQTL data to reveal the protective effect of *GLO1*. However, several limitations should be acknowledged. First, the GWAS data were derived from individuals of European ancestry. As RLS genetic architecture may vary across populations, whether these findings extend to other ancestries remains uncertain. Second, the modest sample sizes of the brain pQTL datasets (ROSMAP, *n =* 376; Banner, *n =* 152) may have limited statistical power to detect weaker associations and could overestimate effect sizes for significant findings, which may partly explain the inconclusive colocalization for *GRWD1* and the TWAS-only signal for *MAP2K5*. Nevertheless, the strong PWAS signals for *GLO1* (*p* = 8.68 × 10^−7^) and *GRWD1* (*p* = 3.59 × 10^−6^) in the Banner dataset suggest adequate power for detecting effects of this magnitude, partially alleviating concerns about insufficient power for these top findings. Third, these brain pQTL data originate exclusively from the dorsolateral prefrontal cortex (dlPFC), which was selected as the most comprehensive available brain pQTL resource, though RLS primarily involves subcortical circuits. Future studies using pQTL data from subcortical regions (e.g., striatum, thalamus) are needed to confirm the regional specificity of *GLO1* associations. Fourth, the predicted drug candidates, including Gambierol, require experimental validation, as their safety, pharmacokinetics, bioavailability, and blood–brain barrier permeability remain unexplored. Fifth, the causal role of *GLO1* in RLS remains to be confirmed by functional experiments. Future studies could test whether *GLO1* activation mitigates RLS-related phenotypes in iron-deficiency-induced neuronal models or established animal models of RLS. Finally, the structural insights from molecular docking are computational and require further chemical optimization and experimental validation to demonstrate clinical efficacy.

## 4. Materials and Methods

### 4.1. Study Design

**Figure 3:**
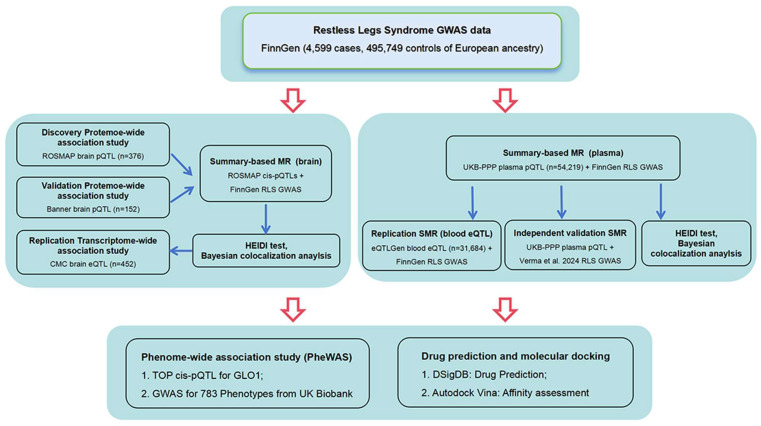
Flowchart of study design.

First, we performed PWAS by integrating RLS GWAS with two brain pQTL datasets to identify RLS-associated proteins. Second, we validated these findings using TWAS, SMR, and colocalization analyses in brain eQTL (CMC) and blood eQTL (eQTLGen) datasets. Third, we further extended validation to the peripheral level using SMR analysis of plasma pQTL data from the UK Biobank (UKB-PPP). Finally, we assessed target specificity via PheWAS and evaluated druggability through drug prediction and molecular docking. A flowchart depicting the methodology used in this study is shown in Figure 3.

### 4.2. GWAS Data of RLS

The datasets used in the current study are listed in Supplementary Table S7. In the main analysis, we examined RLS GWAS data from the Finngen (R12), comprising 4599 cases and 495,749 controls of European ancestry. In the verified analysis, we examined RLS GWAS data from the study by Verma et al. (2024), which consists of 15,475 cases and 428,445 controls of European ancestry [20].

### 4.3. Human Brain Proteome References in PWAS

Two PWAS analyses were conducted using two brain protein quantitative trait locus (pQTL) datasets (discovery and confirmation) to investigate the repeatability and comprehensiveness of the identified proteins associated with RLS. In the discovery dataset, using dorsal lateral prefrontal cortex (dlPFC) tissues from 376 European Ancestry participants, Wingo et al. created the European Ancestry of the Religious Orders Study/Memory and Aging Project (ROSMAP) cohorts [35,36]. The normalized abundance of 8356 proteins was estimated after accounting for clinical features and technical parameters. Among them, 1475 proteins had notable cis-interactions with genetic variation. In this study, 1475 protein weights were used to calculate protein-weighted average scores.

For the confirmation dataset, Wingo et al. produced the Banner Sun Health Research Institute (Banner) dataset using the dlPFC from postmortem brain samples of 152 individuals of European Ancestry [18]. The proteomic analysis approach used for the Banner dataset was identical to that outlined before for the ROSMAP dataset. Of the 8168 proteins that met the quality standards, 1139 were heritable. The weights of 1139 proteins were utilized to validate the PWAS in the replication analysis.

### 4.4. Human Brain Transcriptome References in the TWAS

Transcriptome data were obtained from the dlPFC of postmortem brain tissues collected from 452 people by the CommonMind Consortium (CMC) [37]. More comprehensive information can be found in the original research [37]. A total of 5420 mRNAs were identified as heritable, and their corresponding mRNA weights were obtained from http://gusevlab.org/projects/fusion/ (accessed on 3 May 2026).

### 4.5. FUSION Analysis

The Functional Summary-based Imputation (FUSION) program (Gusev Lab, Harvard T.H. Chan School of Public Health, Boston, MA, USA, http://gusevlab.org/projects/fusion/, accessed on 3 May 2026) was used to calculate protein weights by analyzing proteomic and genomic data from ROSMAP, Banner, and CMC [12]. We utilized FUSION to calculate the impact of single-nucleotide polymorphisms (SNPs) on protein levels for proteins exhibiting substantial heritability (heritability *p* < 0.01). Several prediction models, including top1, blup, lasso, enet, and bslmm, were used in the investigation [12]. The protein weights from the most accurate model were chosen. We used FUSION to integrate the genetic impact of RLS (GWAS z scores) with protein weights by summing z score × weight for each SNP at the locus to construct the PWAS for RLS patients.

### 4.6. SMR Analysis

Summary-based Mendelian randomization (SMR) and heterogeneity in dependent instruments (HEIDI) provide a statistical framework for assessing whether gene expression mediates the effect size of an SNP on a phenotype by integrating with xQTL data [38]. The statistic FDR-corrected *p* < 0.05 was considered significant, and a HEIDI test *p* > 0.05 was considered indicative of independence from LD. To validate our findings, we used three independent datasets: brain eQTL data from the CommonMind Consortium (CMC), blood eQTL data from the eQTLGen consortium, and plasma pQTL data from the UK Biobank Pharma Proteomics Project (UKB-PPP).

### 4.7. Bayesian Colocalization Analysis

We performed colocalization analysis using the coloc R package (version 5.2.3, developed by Chris Wallace, University of Cambridge, Cambridge, UK; available at https://cran.r-project.org/, accessed on 3 May 2026) [39]. Signals from GWASs and pQTLs were associated with SNPs based on five hypotheses (Hx). Null hypothesis (H0): No association with either GWAS or protein quantitative trait loci (pQTLs). Alternative hypothesis (H1): Association with GWASs but not with pQTLs. Alternative hypothesis (H2): Association with pQTLs but not with GWASs. Alternative hypothesis (H3): Association with both GWASs and pQTLs involving two independent SNPs. Alternative hypothesis (H4): Association with both GWASs and pQTLs, including one common SNP. Colocalization uses the Bayesian test to determine the posterior probability of these five hypotheses. A threshold of H4 > 0.80 was used for colocalization analysis.

### 4.8. PheWAS Association Study

To assess potential off-target effects of *GLO1*, we performed a phenome-wide association study (PheWAS) using its two independent cis-pQTLs: rs1781735 (from the ROSMAP brain pQTL dataset) and rs4746 (from the UKB-PPP plasma pQTL dataset). Summary-level GWAS data for 783 diseases (each with >500 cases) were obtained from the UK Biobank (*n =* 408,961), where association tests were conducted using the SAIGE method to account for case–control imbalance [40]. For each phenotype, we extracted SNP-disease association statistics from the SAIGE GWAS database. Statistical significance was defined as Bonferroni-corrected *p* < 0.05. This analysis evaluated the specificity of *GLO1* as a therapeutic target for RLS.

### 4.9. Candidate Drug Prediction

Assessing protein–drug interactions is crucial for determining the feasibility of utilizing target genes as potential drug targets. In this study, we used the Drug Signatures Database [41] (http://dsigdb.tanlab.org/DSigDBv1.0/, accessed on 3 May 2026). Specifically, DSigDB comprises a substantial repository of 22,527 gene sets and 17,389 distinct compounds spanning 19,531 genes. This extensive database serves as a valuable resource for establishing connections between medications, other chemical entities, and their respective target genes. To evaluate their therapeutic potential, drug candidates were predicted by uploading the identified target genes to the DSigDB. Using DSigDB, we hope to gain insights into the medicinal activity of target genes, thereby facilitating the identification of promising drug candidates.

### 4.10. Molecular Docking

Molecular docking provides atomic-level insights into the binding properties of drug candidates and target proteins, enabling the prediction of binding affinities. Binding cavities of the target protein were first predicted using the CB-Dock2 web server [42] (Cao Lab, Central China Normal University, Wuhan, China, http://cadd.labshare.cn/cb-dock2/, accessed on 3 May 2026), which automatically detects potential pockets via its CurPocket algorithm and determines the optimal grid box parameters for docking. The three-dimensional structure of human glyoxalase I (*GLO1*) was retrieved from the AlphaFold Protein Structure Database [43] (https://alphafold.ebi.ac.uk/, accessed on 3 May 2026). The ligand (CID 1567) was obtained from the PubChem Compound Database [44] (https://pubchem.ncbi.nlm.nih.gov/, accessed on 3 May 2026), and its three-dimensional conformation was generated using the Conformer3D module integrated in CB-Dock2.

Based on the cavity with the largest volume (box size = 14 × 14 × 14 Å), molecular docking was performed using AutoDock Vina (version 1.2.0, The Scripps Research Institute, La Jolla, CA, USA) [45] as implemented in the CB-Dock2 server. Default docking parameters were used. The predicted binding affinities (kcal/mol) were used to rank the docking poses. Docking results were visualized directly within the CB-Dock2 output interface, which employs NGL Viewer (developed by Alexander S. Rose et al.; available at https://github.com/arose/ngl, accessed on 3 May 2026) [46] for interactive three-dimensional rendering. The best-scored pose for each pocket was exported and further analyzed in PyMOL (version 3.0, Schrödinger, LLC, New York, NY, USA) [47] to illustrate detailed binding interactions.

### 4.11. Statistical Analysis

All analyses were performed in R (version 4.3.2, R Foundation for Statistical Computing, Vienna, Austria). PWAS significance was determined using Bonferroni correction (*p* < 0.05). For SMR, significance required an FDR-corrected *p* < 0.05 with a HEIDI test *p* > 0.05. For TWAS, FDR-corrected *p* < 0.05 was applied. Colocalization was considered significant at PP.H4 > 0.80, and PheWAS significance was set at Bonferroni-corrected *p* < 0.05. Molecular docking was performed with AutoDock Vina as implemented in CB-Dock2.

## 5. Conclusions

This study identifies *GLO1* as a putative causal gene for RLS, with protective effects mediated by enhanced methylglyoxal detoxification. Convergent multi-tissue evidence, target specificity, and druggability position *GLO1* as a promising candidate for mechanism-based therapy, advancing our understanding of RLS pathogenesis and therapeutic development.

## Figures and Tables

**Table 1:** The discovery in brain PWAS identified 3 significant genes associated with RLS.

Gene	Chromosome	Discovery PWAS (ROSMAP)			Confirmation PWAS (Banner)		
		PWAS.Z	PWAS.P	P_adjust	PWAS.Z	PWAS.P	P_adjust
GLO1	6	−4.903	9.45 × 10^−7^	0.00166	−4.919	8.68 × 10^−7^	0.00099
GRWD1	19	−4.740	2.13 × 10^−6^	0.00375	−4.634	3.59 × 10^−6^	0.00411
MAP2K5	15	/	/	/	6.044	1.50 × 10^−9^	1.72 × 10^−6^

**Table 2:** Risk genes verified by SMR, colocalization analysis and TWAS in brain.

Gene	Outcome Scource	SMR (ROSMAP)						Colocalization Analysis		TWAS (CMC.BRAIN)	
		TOP SNP	Beta	Pval	OR (95%CI)	P_HEIDI	N_HEIDI	PP.H4	H4/(H3 + H4)	TWAS.Z	TWAS.P
GLO1	Finngen_R12_G6_RLS	rs1781735	−2.814	0.0001	0.060 (0.014–0.248)	0.983	12	0.962	0.963	−1.977	0.048
GRWD1		rs2302951	−0.985	0.0062	0.373 (0.184–0.755)	0.343	5	0.517	0.981	−3.464	5.32 × 10^−4^
MAP2K5		/	/	/	/	/	/	/	/	4.388	1.15 × 10^−5^

**Table 3:** The discovery in plasma SMR identified 1 significant gene associated with RLS.

Gene	Exposure Consortium	Outcome Scource	TOP SNP	Beta	Se	OR (95%CI)	Pval	P_Adjust	P_HEIDI	N_HEIDI	COLOC-PP.H4
GLO1	UKBppp (Discovery)	Finngen_R12_G6_RLS	rs4746	−0.389	0.0657	0.677 (0.596–0.771)	3.164 × 10^−9^	5.275 × 10^−6^	0.999	20	5.75 × 10^−6^
GLO1	UKBppp (Confirmation)	Verma et al. 2024 [20]	rs4746	−0.213	0.0354	0.808 (0.753–0.868)	3.162 × 10^−6^	/	0.999	20	5.44 × 10^−6^
GLO1	eQTLgen (Confirmation)	Finngen_R12_G6_RLS	rs1781735	−0.320	0.0657	0.726 (0.639–0.826)	1.117 × 10^−6^	/	0.999	20	2.55 × 10^−27^

**Table 4:** Candidate drugs predicted by DSigDB and their affinities predicted by AutoDock Vina.

Drug Names	Collection	*p*-Value	Adjusted *p*-Value	Affinity (kcal/mol)
2-mercaptoethanol	TTD 00000601	5.9998 × 10^−4^	0.0162	−2.3
Gambierol	CTD 00003965	6.4998 × 10^−4^	0.0176	−8.3
methylglyoxal	CTD 00006677	6.9998 × 10^−4^	0.0189	−6.5
indomethacin	NA	0.0011	0.0311	−6.6
L-methionine	CTD 00006293	0.0012	0.0324	−3.7
FERRIC AMMONIUM CITRATE	CTD 00000709	0.0012	0.0338	−5.0
kaempferol	TTD 00008767	0.0017	0.0459	−7.5

## Data Availability

No new data were generated in this study. All data analyzed are publicly available summary statistics from the following sources: FinnGen RLS GWAS (https://www.finngen.fi/, accessed on 3 May 2026) and Verma et al. [20]; ROSMAP brain pQTL (https://adsp-fgc.niagads.org, accessed on 3 May 2026); Banner brain pQTL (described in Beach et al. [24] and Wingo et al. [22]); CommonMind Consortium (CMC) brain eQTL (http://CommonMind.org, accessed on 3 May 2026); eQTLGen blood eQTL (https://www.eqtlgen.org, accessed on 3 May 2026); UK Biobank Pharma Proteomics Project (UKB-PPP) plasma pQTL (https://registry.opendata.aws/, accessed on 3 May 2026). Supplementary Tables are included with the manuscript.

## Supplementary Materials

The following supporting information can be downloaded at: https://www.mdpi.com/article/10.3390/ijms27104446/s1.
